# High Throughput Sequencing of Extracellular RNA from Human Plasma

**DOI:** 10.1371/journal.pone.0164644

**Published:** 2017-01-06

**Authors:** Kirsty M. Danielson, Renee Rubio, Fieda Abderazzaq, Saumya Das, Yaoyu E. Wang

**Affiliations:** 1 Cardiovascular Institute, Massachusetts General Hospital, Boston, MA, United States of America; 2 Center for Cancer Computational Biology, Department of Biostatistics and Computational Biology, Dana-Farber Cancer Institute, Boston, MA, United States of America; Universidade Federal do Rio Grande do Sul, BRAZIL

## Abstract

The presence and relative stability of extracellular RNAs (exRNAs) in biofluids has led to an emerging recognition of their promise as ‘liquid biopsies’ for diseases. Most prior studies on discovery of exRNAs as disease-specific biomarkers have focused on microRNAs (miRNAs) using technologies such as qRT-PCR and microarrays. The recent application of next-generation sequencing to discovery of exRNA biomarkers has revealed the presence of potential novel miRNAs as well as other RNA species such as tRNAs, snoRNAs, piRNAs and lncRNAs in biofluids. At the same time, the use of RNA sequencing for biofluids poses unique challenges, including low amounts of input RNAs, the presence of exRNAs in different compartments with varying degrees of vulnerability to isolation techniques, and the high abundance of specific RNA species (thereby limiting the sensitivity of detection of less abundant species). Moreover, discovery in human diseases often relies on archival biospecimens of varying age and limiting amounts of samples. In this study, we have tested RNA isolation methods to optimize profiling exRNAs by RNA sequencing in individuals without any known diseases. Our findings are consistent with other recent studies that detect microRNAs and ribosomal RNAs as the major exRNA species in plasma. Similar to other recent studies, we found that the landscape of biofluid microRNA transcriptome is dominated by several abundant microRNAs that appear to comprise conserved extracellular miRNAs. There is reasonable correlation of sets of conserved miRNAs across biological replicates, and even across other data sets obtained at different investigative sites. Conversely, the detection of less abundant miRNAs is far more dependent on the exact methodology of RNA isolation and profiling. This study highlights the challenges in detecting and quantifying less abundant plasma miRNAs in health and disease using RNA sequencing platforms.

## Introduction

Extracellular RNAs (exRNA) have recently been identified as novel biomarkers and potential cell-cell communicators in plasma and other body fluids. To date, the most abundantly studied class of these exRNAs are microRNAs (miRNA), which have been implicated in a wide range of diseases including heart failure [1, 2], cancer [3, 4], and multiple sclerosis [5, 6]. miRNAs are small, non-coding RNAs that have the ability to regulate whole networks of genes through transcriptional and translational regulation. In the circulation they are commonly found enclosed in extracellular vesicles (EVs) [7], bound to lipoproteins [8], or complexed with Argonaute-2 [9]. Furthermore, *in vitro* studies have shown that numerous cell types are capable of releasing miRNA in these complexes. exRNA profiling may therefore reflect cellular content and identify disease specific variations in expression. More recently, the application of next generation sequencing to exRNA discovery has led to the recognition that various other species of RNAs are also present in biofluids [10, 11]. While not as extensively studied as miRNAs, emerging data suggests a role for these other species of RNA as prognostic biomarkers in disease [12], and as possible mediators of disease pathogenesis.

While exRNA offers an exciting new branch of functional biomarker research, the field is still in its infancy and faces numerous technical challenges. RNA concentrations in biofluids are significantly lower than in tissue and standard methodologies developed for tissue RNA extraction and quantification are not always appropriate. Secondly, the RNAs may be present in different compartments, each of which may have different susceptibilities and vulnerabilities to RNA isolation techniques, thereby creating an unintended bias in RNA profiling based on the exact isolation method used [13, 14]. Furthermore, samples available to researchers for study have often not been collected for the purpose of exRNA analysis and have been archived for significant lengths of time. Finally, little is known about the normal physiology of exRNAs and what level of variation exists between healthy donors. These factors all need to be taken into consideration in the development of standard protocols for the study of exRNA. Recent studies have compared different commercially available RNA isolation kits in their relative efficiencies in isolating RNA from plasma [13] or EVs derived from plasma [11]. Based partly on these results, and partly on our preliminary experiments, we chose the miRCURY RNA Isolation Kit for Biofluids (Exiqon) as the standard kit in this study to address other variabilities in RNA isolation with small RNA sequencing as the ultimate output.

Current technologies available for the quantification of exRNA include qRT-PCR, RNA microarray, and RNA sequencing (RNAseq). While qRT-PCR and RNA microarrays utilize specific primers or probes, and as such can only detect known RNA sequences, high through-put RNAseq has the ability to detect novel transcripts across a broad dynamic range and offers a potentially sensitive means to characterize and quantify exRNA. However, in addition to the biases introduced by the technique [11, 15], the landscape of exRNAs in biofluids such as plasma appear to be dominated by certain species of RNA, such as ribosomal RNA fragments or particular miRNAs: this leads to a skewed distribution of exRNA species which limits the sensitivity of detection of less abundant species. This study aims to address some of the current caveats for RNAseq, specifically by optimizing aspects of exRNA extraction from plasma for the quantification of miRNA by RNAseq.

## Materials and Methods

### Subjects

All experiments were conducted with approval of the Institutional Review Board of Beth Israel Deaconess Medical Center and written consent of the subjects. Blood was obtained from 4 healthy adult volunteers (2 males, 2 females) between the ages of 20–40 for all experiments except the long archived ribodepleted vs non-ribodepleted samples. These samples were obtained from a registry of post-myocardial infarction patients with collected plasma for assay of novel biomarkers (PROSPECT-CMR). These patients (2 female, 1 male) were between the ages of 49–70, where Pt 1 and Pt 3 are females and Pt 2 is male.

### Plasma Isolation

40 mL of blood was obtained by venipuncture using a 21G needle and collected in K_2_EDTA-containing tubes. Blood was immediately processed by centrifugation at 1,000 x g for 10 min at room temperature to separate plasma. The isolated plasma was immediately frozen at -80°C in 1 mL aliquots until use. Samples were frozen for approximately 1 week prior to extraction, except where noted. Plasma was thawed at room temperature and centrifuged at 2,000 x g prior to RNA extraction to remove any remaining platelet or debris contamination.

### RNA Extraction and Treatment

RNA was extracted from 1 mL of plasma using the miRCURY RNA Isolation Kit for Biofluids (Exiqon) according to manufacturer’s protocol except for noted modifications. 1 μL of 20 mg/mL glycogen (Roche) was added to plasma prior to RNA extraction. All samples were treated with T4 polynucleotide kinase (New England Biolabs) to facilitate 5’ hydroxyl terminus phosphate labelling and allow greater binding of adaptors during library preparation, and RNA sample volume was reduced to ≤10 μL in a standard lyophilizer at room temperature. RNA concentration was determined by Quant-iT RiboGreen RNA Assay Kit.

**Fig 1:**
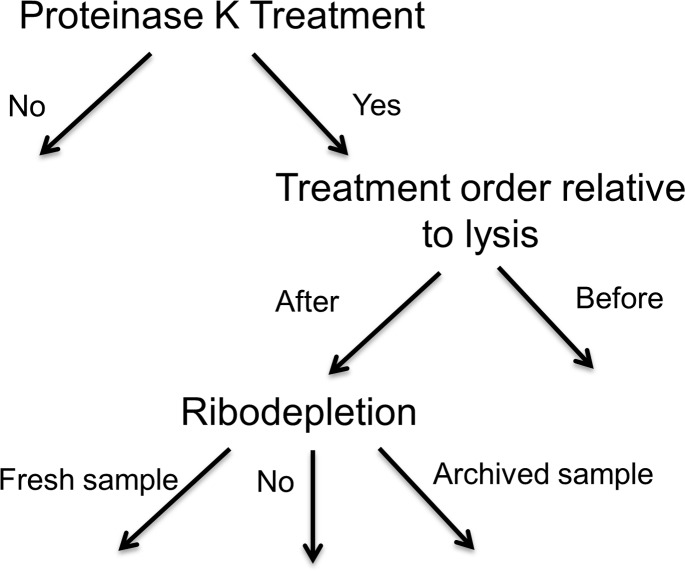
Schematic summarizing plasma sample treatments used in this study. RNA isolation was performed with or without proteinase K treatment and ribodepletion on fresh or archived samples.

Prior to RNA extraction, a subset of plasma samples were treated with Proteinase K (PK; 100 μg/mL in 0.5% SDS solution) for 30 min at 50°C. Samples were treated in one of three groups: 1) no PK treatment; 2) PK treatment prior to addition of guanidinium thiocyanate (GITC) containing lysis buffer (from miRCURY RNA Isolation Kit); 3) PK treatment following the addition of GITC-containing lysis buffer. Where indicated, ribodepletion was performed using the Ribo-Zero Magnetic Gold kit (Epicentre) according to manufacturer’s protocol. A schematic summarizing plasma sample treatments is shown in Fig 1.

For samples that were analyzed by digital droplet PCR (ddPCR), ethanol precipitation was performed to remove any excess SDS solution that could inhibit the PCR reaction. Briefly, 0.3 M NaCl, and 3x volumes of 100% ethanol were added to the solution and the RNA precipitated at -80°C overnight. Samples were then pelleted by centrifugation, washed with 80% ethanol, and re-suspended in 10 μL RNAse-free water.

### RNA Library Preparation

Each sequencing library was constructed from 2 ng of isolated and treated plasma RNA. All libraries were uniquely bar-coded with index primer for multiplexing into sequencing lanes. The small RNA libraries were prepared and amplified using the NEBNext small RNA Library Prep Set (New England BioLabs, Ipswitch, MA, USA) following manufacturer instruction. The amplified libraries were resolved on a 10% Novex TBE gel (Life technologies) for size selection and the 140 to 160 nucleotide bands that correspond to adapter-ligated constructs derived from the 21 to 40 nucleotide RNA fragments were excise and recovered in DNA elution buffer. The average size distribution of each library was determined using Agilent Bioanalyzer with High Sensitivity Chip Kit (Agilent, Santa Clara, CA, USA) and quantified on ABI 7900HT Fast RT-PCR instrument using the KAPA Library Quantification kit according to the manufacture’s protocol (Kapa Biosystems, Woburn, MA, USA). Each library was adjusted to final concentration of 2 nM, pooled, and sequenced on an Illumina HiSeq 2000 or MiSeq sequencer for single read 50 cycles at the Center for Cancer Computational Biology at Dana-Farber Cancer Institute.

### Sequence and Statistical Analysis

The BCL files were de-multiplexed using CASAVA v1.82, and the adaptor sequences within the read sequences were trimmed by FastX-Toolkit (*http://hannonlab.cshl.edu/fastx_toolkit*). The processed sequences were filtered for small RNAs greater than 16 nucleotides in length. The sequences were then aligned, quantified and annotated using sRNABench 1.0 pipeline [16]. Briefly, the pipeline implemented hierarchical sequence mapping strategy that first mapped and remove spike-in library, contaminants, and rRNA before sequentially mapped to known mature miRNA, tRNA, snoRNA and piRNA onto the human genome sequence (hg19) using Bowtie2 [17] with parameters that allow for 1 mismatch in seed alignment (-N 1), try two set of seeds (-R 2), and set the length of seed substrings to be 16 (-L 16). Mapped small RNA species was quantified to read counts and normalized to RPM as described in sRNABench. Detected species were mapped to mature miRNA only and not precursor miRNA. Reads derived from microRNA with multiple copies in the genome were summed together, and read counts from sample duplicates were aggregated by mean for where it is applicable. All statistical analysis was performed using R version 3.2. To avoid introducing inherent variability by normalization methods for miRNA [18, 19], only read counts were used for Spearman correlation analysis. All fastq files had been deposited into the exRNA Atlas (http://exrna.org/resources/data/) as part of Extracellular RNA Communication Consortium (ERCC).

### Digital Droplet PCR

RNA was diluted 1:10 in RNAse-free water and reverse transcribed with the Universal cDNA synthesis kit II (Exiqon) according to manufacturer’s protocol. cDNA was diluted 1:10 and loaded into PCR reactions with EvaGreen supermix (Bio-Rad) and pre-designed primers for miR-30d, miR-150, and miR-122 (Exiqon) according to manufacturer’s protocol (Exiqon). Droplet formation was carried out using a QX200 droplet generator and droplets were transferred to a 96-well plate for PCR. No template reactions were used as a negative control. Endpoint amplification was conducted with the following steps: 95°C for 5 min, 40 cycles of 95°C for 30 s, 58°C for 30 s, 4°C for 5 min and 90°C for 5 min at a ramp-rate of 2.5°C/s for all steps. Droplets were then read with the QX200 droplet detector (Bio-Rad) and analysis conducted with Quantsoft 1.7. Results are presented as expression relative to the no PK treatment counts.

## Results

### Proteinase K treatment after lysis achieved highest yield of microRNA

To determine the impact of proteinase K (PK) treatment at different stages of RNA extraction affecting miRNA detection, we extracted RNA from plasma treated with **a)** no PK, **b)** PK treatment before GITC (Pre-GITC), and **c)** PK treatment in GITC (PK in GITC and PK in GITC #2) that was drawn from a single subject. The no PK, PK pre-GITC, and PK in GITC samples were from the same blood draw, whereas the PK in GITC #2 was from a separate draw. Total RNA yield from these samples (1 mL starting volume; measured by RiboGreen RNA Assay kit) was a) 22.1 ± 1.3 ng, b) 23.0 ± 3.2 ng, and c) 67.0 ± 6.0 ng (mean ± SEM). Libraries were constructed using NEBNext small RNA Library Prep Set and run on the Illumina HiSeq2000 platform. The number of initial reads post-filtering varies from 5.5 to 8.4 million for each sample, with more than 50% of the reads being ribosomal RNA (rRNA) (Table 1). The rRNA reads were first filtered and the remaining reads were mapped against the human genome (hg19) and 2578 annotated mature miRNA sequences. We observed 43.3%, 27.9% and 32.1% of reads mapped to human genome for no-PK, Pre-GITC, and PK-in-GITC samples respectively; however, the percentages of miRNA reads were the lowest for the no-PK sample at 3.7%.

**Table 1:** Read counts in all samples analyzed.

	Subject	Sample	# Total Reads	# rRNA read	% rRNA	# non-rRNA mapped reads	Non-rRNA mapping rate**	# miRNA Reads	miRNA mapping rate **	# mature miRNA detected
	1	no PK	5574492	3734826	67.0%	796358	43.3%	29354	3.7%	358
		PK pre-GITC	8405768	5860872	69.7%	710916	27.9%	74175	10.4%	346
		PK in GITC	6504050	3481787	53.5%	971510	32.1%	119100	12.3%	428
32A		PK in GITC #2	14086341	9899542	70.3%	1310291	31.3%	192574	14.7%	317
		RD1 (Fresh)	31218443	7805259	25.0%	7392722	34.3%	3224683	43.6%	584
		RD2 (Fresh)	35787174	5355049	15.0%	12790223	43.4%	6579789	51.4%	645
		RD1 (Archived)	6903917	4469521	64.7%	1223479	59.5%	1077	0.1%	97
		RD2 (Archived)	5041307	3466070	68.8%	698977	53,11%	124	0.0%	61
	Pt1	No RD	24521036	8546616	34.85%	3641826	36.10%	106480	2.92%	179
		RD	31521362	19250442	61.07%	3848929	47.71%	93	0.00%	51
	Pt2	No RD	22661624	10038903	44.30%	2181962	23.96%	94197	4.32%	276
		RD	16452990	12744367	77.46%	872479	78.68%	841	0.10%	66
	Pt3	No RD	17221688	6629892	38.50%	2531001	35.34%	223791	8.84%	280
		RD	22892313	15538787	67.88%	2052797	47.11%	77	0.00%	48
	2*	a	24103148	18769039	77.9%	533392	66.6%	68102	12.8%	318
		b	24544904	15020704	61.2%	1254211	64.0%	84332	6.7%	409
	3*	a	12061323	8163497	67.7%	557689	70.8%	23841	4.3%	298
		b	46331162	28588507	61.7%	1363618	70.6%	166393	12.2%	504
	4*	a	28194355	282050	1.0%	407866	4.5%	16960	4.2%	216
		b	41819930	945503	2.3%	527795	4.7%	12803	2.4%	215

* RNA isolated with PK in GITC. Sample a and b are duplicates from the same blood draw.

** mapping rate after removal of rRNA sequences.

Abbr: Proteinase K (PK), guanidinium thiocyanate(GITC), Ribodepletion (RD).

**Fig 2:**
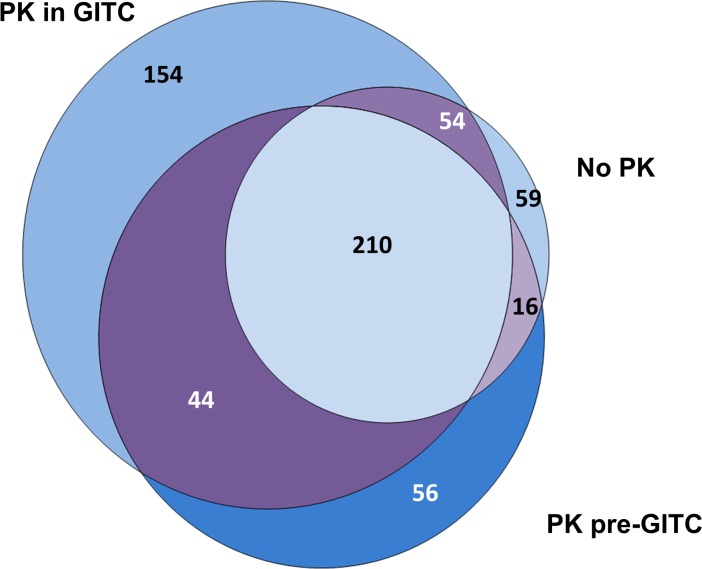
Venn diagram of mature miRNA species detected in each treatment group. Plasma samples libraries from a single health donor generated using no PK, PK treatment before GITC, PK treatment in GITC show strong concordance of miRNA species detected, with PK treatment in GITC method showing higher sensitivity than others.

Despite the large variation in the total number of miRNA reads, all three samples yielded more than 346 mature miRNA with PK-in-GITC sample yielding the most diverse result (n = 428). Overall we identified reads mapping to 593 mature miRNA across samples with detectable counts (count>0, S1 Table), but only 210 out of 593 miRNA were shared among them with modest level of correlation (r>0.43, Spearman Correlation, Fig 2), suggesting strong miRNA profile variability as a result of different PK treatment. If only miRNA with >10 reads were considered, the number reduced to 97 shared and 198 total mature miRNA. The degree of correlation also showed general increases between all protocols (r>0.67), with Pre-GITC and PK-in-GITC samples showing the highest correlation (r = 0.75).

Lastly we checked if commonly observed miRNA species were expressed at higher levels and thus more resistant to technical variation. miRNA that were observed in all samples showed significantly higher expression compared to those observed in two or less samples (P<2.09e-05, Wilcoxon Rank Sum Test). Similarly, miRNAs uniquely observed in only one sample generally have lower expression, particularly among those uniquely identified from the PK in GITC sample. We repeated PK-in-GITC treatment on a different plasma sample (PK-in-GITC #2) derived from the same subject, and observed similar level of percentage mapped reads (31.3%, Table 1). Taken together, this suggests PK in GITC resulted in higher RNA yield as well as higher miRNA sequencing sensitivity.

To further validate these findings we performed ddPCR on repeated samples of no PK vs PK in GITC from the original single healthy donor. From the RNAseq results, we selected a high abundant (miR-122), mid-abundant (miR-30d), and low abundant miRNA (miR-150) species to measure (S1 Fig). Interestingly, the greatest increase in species count with PK in GITC treatment was seen in the low abundant miR-150 both with RNAseq (PK in GITC vs No PK: 40 fold change) and with ddPCR (PK in GITC vs No PK: 1.13 fold change). A modest increase was seen in miR-30d: 2.9 fold by RNAseq and 1.09 fold by ddPCR. Finally, the high abundant miR-122 showed a 1.4 fold increase by RNAseq and a 0.81 fold decrease by ddPCR. This suggests that PK treatment increases availability and yield of low abundant miRNA, whereas the high abundance species may be at a saturated detection level that is not affected so greatly.

### The Effect of Ribodepletion on miRNA Detection

Due to the high percentage of rRNA reads observed, the effect of ribodepletion on sample reads was tested. Plasma samples from the same healthy individual, frozen for either 1 week (fresh) or 1 year (archived) were extracted with PK pre-treatment and ribodepletion was performed. As expected, ribodepleted fresh samples showed decreases in the percentage of rRNA reads, ranging from 15 to 25%, while the percentages of miRNA reads and detectable mature miRNA species both showed increases (Table 1) when compared to non-ribodepleted samples. However, ribodepletion of the archived samples did not result in a decrease of rRNA reads. Instead, the ribodepletion step appeared to significantly reduce the percentages of miRNA reads, with the number of detected miRNA species decreased to less than a hundred in both samples (Table 1). The degree of expression correlation also weakened substantially among the commonly identified miRNA species (r = 0.43 for RD1, r = 0.27 for RD2, Spearman’s Test, S2 Fig).

To further validate this observation, we sequenced both ribodepleted and non-treated archived plasma samples derived from three cardiovascular patients (subjects Pt1 to Pt3) that were archived for 3–4 years. The result concurred with previous observation. Ribodepleted archived samples consistently yield dramatically lower percentage of miRNA mapping reads and lower number of miRNA species compare to non-treated samples (Table 1, S2 Table). Paradoxically, the percentages of ribosomal reads were higher among the ribodepleted samples. As most samples from clinical settings would have been frozen for extended period of time, ribodepletion was not incorporated for rest of this study despite the observed improvement in fresh plasma samples.

### Variability of miRNA Expression Between Samples

To assess technical reproducibility of miRNA profiling, we examined duplicated miRNA profiles derived from the plasma of three healthy subjects between the ages of 20 to 40 (raw miRNA read counts in S3 Table). We obtained greater than 12 million reads per sample, but again with more than 60% being ribosomal RNA in subject 2 and 3. While samples derived from subject 4 exhibited lower percentage of rRNA reads, these samples contained high level of adaptor dimers and mapped poorly to the human genome (4.5% of total raw reads). An average of 326 known miRNAs were quantified, with subject 4 samples showing the lowest number of miRNA diversity.

**Fig 3:**
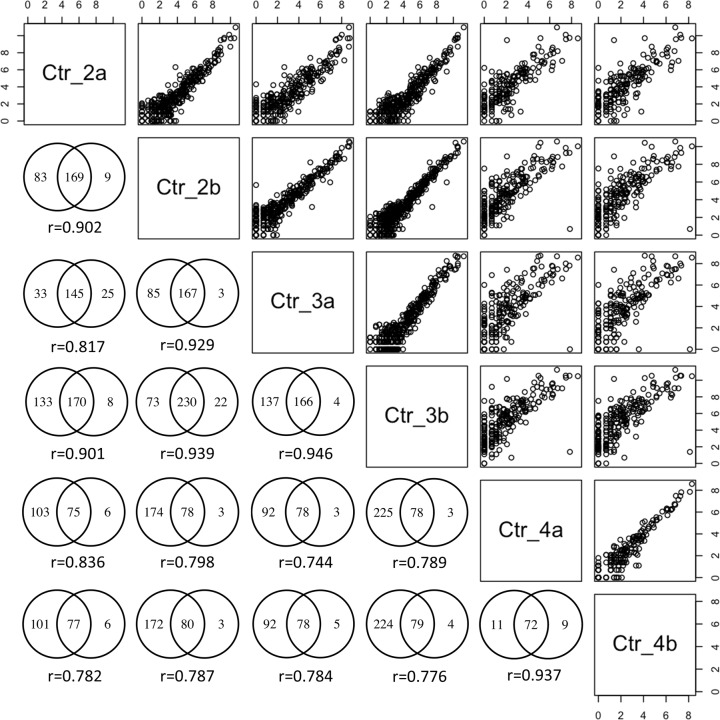
Spearman correlation for the expression level of detected microRNAs. While different numbers of miRNAs were quantified between duplicated samples, the majority of these miRNA were commonly observed. The expression level of these commonly observed miRNA were highly correlated for both duplicated samples (r>0.9) and across samples from different subjects (r>0.74).

To determine sequencing reproducibility, we compared quantified miRNA species between sample duplicates. We considered all quantifiable miRNA from individual run with read count of greater than 10 to minimize sampling fluctuation. The overlaps between biological duplicates were modest, ranging from 54% to 63% of all quantified miRNA across duplicates (Fig 3). We then determined if the overlapping miRNA species were more highly expressed and thus likely more resistant to sampling fluctuation by comparing the expression level of commonly observed miRNA (n = 62) across all runs to others. As expected, the expression of commonly observed miRNA species was significantly higher (P<0.001).

To examine whether commonly detected miRNA expression is generally reproducible, we first determined the correlation of these miRNA expression among duplicates. Intra-sample correlations were significant across all samples (P<1e-29, Spearman correlation, Fig 3) with r-values from 0.82 to 0.92, indicating strong expression reproducibility. We then performed the same analysis across all sample pairs to assess miRNA expression concordance across different control samples. The percentages of common miRNA across different control samples were similar to biological duplicates, ranging from 54% to 63% of all quantified miRNA. While the concordances across samples were lower than biological duplicates, the expression levels remain highly correlated with r>0.71 (Fig 3). Notably, samples 4a and 4b, which are the ones with the lowest sequence depth and number of mature miRNA species detected, showed the lowest degree of concordance with other samples. Taken together, plasma miRNA derived from health subjects showed highly consistent expression level across runs and individuals.

### Comparison with Published Data

The expression consistency of plasma miRNA across different individuals suggests there maybe sets of plasma miRNA species that are more conserved in their expression in healthy subjects. Here, we used 142 miRNA (S4 Table) that were expressed in all three healthy subjects and their duplicates as our set of conserved plasma miRNA. Of these, the top 20 expressed miRNA and their read counts in the duplicated samples are shown in Table 2. These include known disease associated miRNA species such as miR-451a [20, 21], miR-1246 [22], miR-423 [23], and miR-148a [24, 25] (Table 3). Overall, these miRNA were significantly highly expressed (P<1.89e-13, Wilcoxon Rank Sum Test) compared to the remaining detected species across all samples.

**Table 2:** Top 20 miRNA by read counts in duplicated control samples.

miRNA	Ctr_2a	Ctr_2b	Ctr_3a	Ctr_3b	Ctr_4a	Ctr_4b
hsa-miR-122-5p	58455	39046	5929	76977	783	912
hsa-let-7a-5p	17066	10828	2482	37568	2472	1551
hsa-let-7b-5p	24642	18992	3010	37999	1747	1418
hsa-miR-451a	16128	22759	5245	35800	5305	3996
hsa-miR-1246	16500	33752	6277	28546	172	61
hsa-let-7f-5p	14536	10490	1789	29832	1789	962
hsa-miR-21-5p	6100	21638	5874	37552	144	113
hsa-let-7i-5p	5464	7009	2311	15237	527	474
hsa-miR-423-5p	3600	6801	1773	8429	833	852
hsa-let-7c-5p	13071	7281	1681	25635	8	4
hsa-miR-148a-3p	4306	5571	2440	13679	495	424
hsa-miR-26a-5p	2443	7606	1906	16809	98	136
hsa-miR-486-5p	3436	5260	2268	14609	191	127
hsa-let-7g-5p	2818	3654	1087	9529	561	429
hsa-miR-101-3p	2431	4659	1193	7285	422	289
hsa-miR-22-3p	1219	5564	3805	9898	127	129
hsa-miR-151a-3p	2410	5172	1872	7694	87	54
hsa-miR-92a-3p	1949	3774	1005	7187	302	457
hsa-miR-126-3p	1014	4021	659	8671	191	177
hsa-miR-320a	958	3623	1631	3743	542	694
hsa-miR-144-3p	1150	2329	746	8285	1259	1209

**Table 3:** Top expressing miRNA with target genes and reported dysregulation in human disease.

miRNA	Target Gene Examples	Reported Dysregulation in Human Disease (top hit on PhenomiR [26])
hsa-miR-122-5p	CCNG1 [27]; IGF1R [28]; AKT3 [29]	Hepatocellular carcinoma [27, 30, 31]
hsa-miR-451a	CAB39 [20]; IL6R [32]; RAB14 [33]	Glioblastoma multiforme [20, 21]
hsa-let-7b-5p	TUBA1C [34]; TAB2 [34]; GATA6 [34]	Acute lymphoblastic leukemia [23, 35, 36]
hsa-let-7a-5p	AGO1 [37]; LARP1 [34]; HMGA2 [38]	Lung cancer [39–41]
hsa-let-7f-5p	SUOX [42]; ZNF8 [43]; EDN1 [43]	Hepatocellular carcinoma [27, 44, 45]
hsa-let-7i-5p	ACOT9 [43]; SLC10A7 [42]; BMP4 [46]	Melanoma [47, 48]
hsa-miR-1246	CTC1 [42]; PYGO1 [49]; HTR7	Stroke [22]
hsa-miR-21-5p	PITHD1 [43]; CDC25A [50]; PTEN [51]	Pancreatic cancer [24, 52, 53]
hsa-miR-423-5p	TACR3 [43]; PABPC1 [34]; VAC14 [34]	Acute lymphoblastic leukemia [23]
hsa-miR-148a-3p	MAP3K4 [34]; CCKBR [54]; DENR [34]	Pancreatic cancer [24, 25, 55]
hsa-miR-486-5p	ABCF2 [49]; H3F3B [43]; PCCA [56]	Glioblastoma multiforme [57]
hsa-miR-26a-5p	PNMA2 [34]; CREBZF [42]; SLC7A1 [58]	Prostate cancer [53, 59]
hsa-let-7g-5p	GAB2 [60]; ATG9A [43]; AKAP8 [42]	Hepatocellular carcinoma [30, 31, 44]
hsa-miR-22-3p	BMP7 [61]; FKBP5 [34]; STX4 [34]	Hepatocellular carcinoma [27, 62]
hsa-miR-101-3p	GLRX5 [42]; RAP1B [63]; EIF4G2 [42]	Leukemia [64–66]
hsa-miR-144-3p	ATXN1 [42]; FGB [67]; MKLN1 [43]	Ovarian cancer [68–70]
hsa-miR-320a	ZEB2 [34]; CLUH [34]; IGF1R [71]	Ovarian cancer [68, 72]
hsa-miR-92a-3p	PAIP1 [42]; MYO1D [58]; GAPDH [34]	Leukemia [36, 66, 73]
hsa-miR-126-3p	VEGFA [42]; PIK3CG [74]; IRS1 [75]	Lung cancer [39, 41]
hsa-miR-103a-3p	KIF23 [42]; HPRT1 [76]; GGA3 [76]	Medulloblastoma [77, 78]
hsa-miR-151a-3p	RYBP [56]; MAP1B [76]; MLH3 [34]	Hepatocellular carcinoma [45, 79]

**Fig 4:**
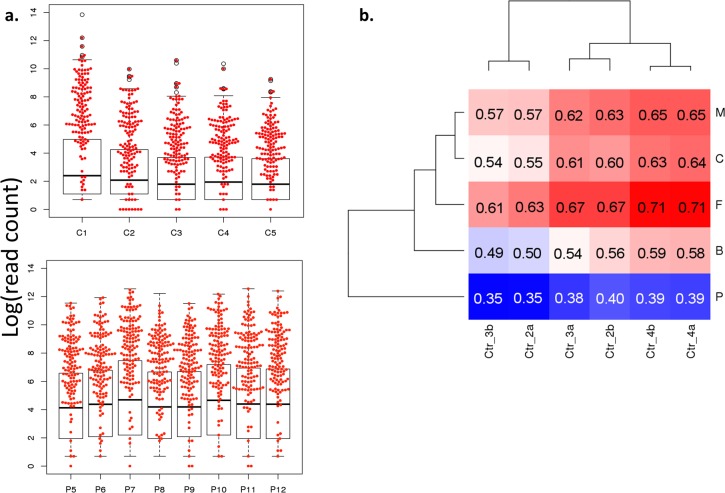
Comparison of the 142 miRNA that were commonly observed across control duplicates against published mother plasma (M), father plasma (F), fetal plasma (B), placenta (P), control female plasma (C) miRNA results. **a)** These miRNA were found to be significantly enriched among the highly expressed miRNA across all sample types (M, F, B types not shown). **b)** The correlation of these miRNA expression were high among the plasma samples (M, F, B, C), but low compare to placenta (P).

In order to assess whether these conserved miRNAs exhibit similar expression patterns in plasma derived from normal subjects and can be detected in other dataset, we compared our sequencing data against plasma miRNA sequence data previously published by *William et al* [80] that was generated with different RNA isolation and library preparation protocols. Briefly, the study sequenced microRNA from plasma collected from mothers, fathers, umbilical cords, and biopsy samples of the corresponding placentas as well as non-pregnant control females in good health. First, we evaluated the expression level of our conserved miRNAs in relation to rest of the detected miRNA in each sample type. While a few of these conserved miRNAs were not observed in all samples, the observed miRNAs were significantly highly expressed compared to rest of the miRNA in all sample types (Fig 4A), including placentas, suggesting these conserved miRNAs that we found were highly expressed in different plasma types and tissue type.

Next we evaluated if these conserved miRNA expression patterns were consistent between the plasma samples from the two datasets. Correlations across datasets for plasma samples were strong (Fig 4B) for plasma samples collected from mothers, fathers, and non-pregnant controls, with most of the paired comparisons with *r* > 0.6 (Spearman Correlation). Plasma samples collected from umbilical cords showed slightly weaker correlation. On the other hand, correlations between placenta and plasma data were generally weaker (P<0.0005, r<0.4) despite showing similar high miRNA expression level among the conserved miRNAs as other sample types. This data indicates that there may exist conserved miRNA expression profiles that are specific to plasma.

### sRNA Content in Plasma by Transcriptome Sequencing

**Fig 5:**
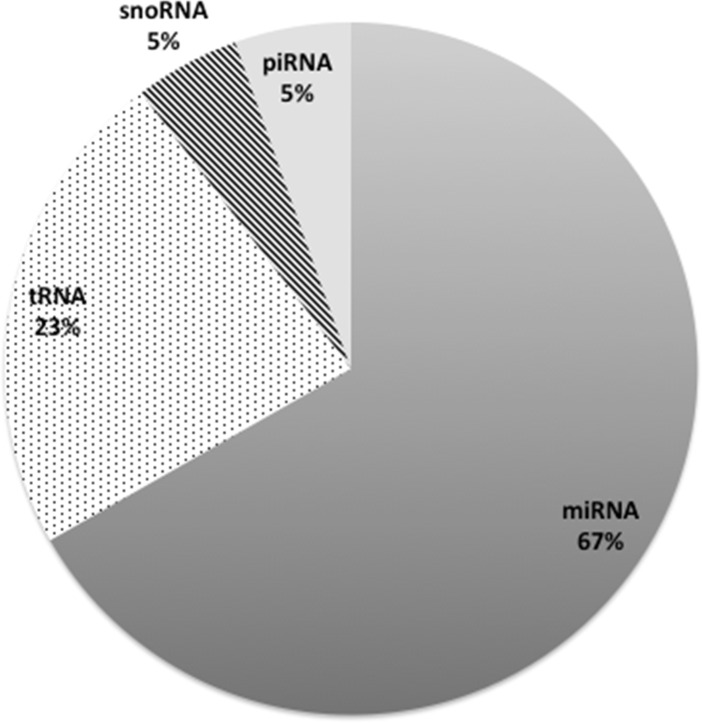
Distribution of characterized plasma small RNA reads across miRNA, tRNA, piRNA, and snoRNA.

We examined the number of reads that were mapped to other class of small RNAs in addition to rRNA and miRNA. Overall, among the total 558,000 reads of small RNA sequences generated from the three healthy subjects, there were 67% miRNA, 23% tRNA, 5% small nucleolar RNA (snoRNA), and 5% piwi-interacting RNA (piRNA) reads mapped (Fig 5). Despite significant amounts of variability in the number of initial and post-processed reads across individual samples, miRNA and tRNA reads were consistently the most and the second most abundant small RNA class identified. Out of the 356 distinct tRNA that were identified, more than half (238 out of 355) were overlapping among the control subjects. The number of overlapping tRNA remained close to half (168 out 355) even after restricting to ones with at least 10 reads (S3 Fig).

## Discussion

We began this study with the intention to establish a robust small RNA extraction procedure for peripheral blood plasma miRNA quantification that can be reliably utilized for biomarker discovery from clinical samples. While settling on a single kit based on other studies, our goal was to examine the effects of modifications of RNA extraction procedures for assessing plasma miRNA expression profiles using NGS as our quantification assay. The experiment was designed to select the optimal RNA extraction procedure on plasma samples derived from a single healthy individual, taking consideration of sample storage duration, and then extended to multiple individuals as well as published data to evaluate consistency.

We first evaluated the impact of PK treatment, which has been reported to increase RNA yield [81], on extracting RNA from plasma. We found that PK treatment following addition of GITC-based denaturing buffer to plasma markedly increases RNA yield, the number of mapped miRNA reads, and improved detection of low-abundance miRNA species. It is known that miRNAs in the circulation are relatively sequestered: associated with EVs, bound to lipoprotein, or complexed with RNA binding proteins. At the same time, RNAses in plasma are relatively more resistant to PK digestion compared to other RNA binding proteins. We therefore think that the timing of addition of PK to the sample is of great importance: PK added directly to the sample may destroy the protective RNA binding proteins more readily than the RNAses, thereby allowing for degradation of exRNAs and lower yields. In the presence of denaturing buffer, the RNAses are inactive (while the PK may be still be active), and the improved RNA yield is presumably due to the dissociation of exRNAs from their RNA binding partners. This may also explain why the low-abundance miRNA are more readily detected following PK in GITC treatment, as confirmed by our ddPCR experiment. We therefore recommend that for discovery purposes PK in GITC treatment is the optimal condition as it allows for detection of a greater number of miRNA species. This may not be necessary for validation studies or where high abundant miRNA is being assessed.

One of the most surprising outcomes from our sequencing data was the consistently high percentage of ribosomal RNA reads that occupies large proportion of sequence reads. This made it more challenging to achieve a high depth of coverage for profiling miRNA expression. We tested the ability of ribodepletion, which is standardly performed for RNAseq of tissues samples, to remove rRNA from extracted plasma RNA derived from fresh peripheral blood plasma. The result was initially highly encouraging with both substantial decrease in the percentage of rRNA and increase in the percentage of miRNA reads. However, the same plasma sample that were frozen for one year yielded poor sequencing results after ribodepletion, with no reduction in the percentage of rRNA and substantial decrease in the percentage of miRNA reads. As most clinical samples would have been archived for extended period of time, it is important to note that fresh and archived samples should be handled differently. The use of ribodepletion, while potentially beneficial in fresh samples, may not be suitable for the study of archived samples. Other techniques, such as blocking of highly abundant miRNAs for plasma RNA sequencing, have been described but not adapted to multiplexing and could offer another avenue of increasing the detection of less abundant species of exRNAs.

Our comparisons of miRNA expression profiles showed strong correlation of miRNA species for both sample replicates and between samples from different healthy donors, supporting the reproducibility of NGS as a methodology for plasma miRNA quantification. In particular, despite the low RNA concentration within peripheral blood plasma that may introduce strong sampling bias, large numbers of abundantly expressed miRNA species were observed across samples that we examined in this study with similar profiles. We further investigated if these conserved plasma miRNA that we observed exhibit similar patterns from healthy individuals in published study and found that these profiles remain strongly correlated with our results despite differences in sample acquisition, RNA extraction, and sequence library preparation protocols. While this may suggest the presence of a stable landscape of extracellular miRNA in healthy plasma, further studies with larger cohorts are required to confirm this observation.

As the scope of this study is limited to optimize only small aspects of exRNA extraction process from plasma for small RNAseq quantification, there remain arrays of challenges in generating reproducible and reliable data. For instance, T4 polynucleotide kinase treatment during sequencing library generation likely facilitated extracellular DNA nucleotide for sequencing [82] to result in DNA sequence contamination in our data. Also, it has been reported that RNA ligases have strong sequence specific biases [83] and can distort small RNA expression profile considerably. While pooled and randomized adapter strategies have been proposed to reduce ligation bias, the technology would need to be further evaluated. The dominance of ribosomal RNA sequence in plasma remains a challenge that constrained our ability to obtain desirable sequencing depth to consistently detect less abundant small RNA species. Overall, this study optimizes exRNA extraction process for plasma small RNA sequencing and further demonstrates NGS platform as a reliable methodology for plasma extracellular biomarker discovery.

## Supporting Information

S1 Table Raw microRNA read counts for plasma sample derived from health subject. Plasma was treated with a) no proteinase K(No_PK); b) PK treatment before GITC (PK_pre_GITC); c) PK treatment in GITC (PK_in_GITC); d) Repeated PK treatment in GITC (PK_in_GITC_2)
(XLSX)

S2 Table Raw microRNA read counts for archived plasma samples with and without Ribodepletion treatment. (XLSX)

S3 Table Raw microRNA read counts for plasma sample derived from three health subject with duplicates. (XLSX)

S4 Table The conserved set of 142 plasma miRNA that were expressed in all three healthy subjects and their duplicates. (XLSX)

S1 Fig Relative expression of miR-122, miR-30d, and miR-150 by RNAseq and ddPCR. (TIF)

S2 Fig Comparison of microRNA expression correlation between two pairs of the same fresh and archived frozen plasma samples after ribosomal depletion treatment. (TIF)

S3 Fig Unique and overlapping tRNA detected from each healthy subject. (TIF)
