# Supplementary figures and images for: High Throughput Sequencing of Extracellular RNA from Human Plasma
Source: PLoS One. 2017 Jan 6;12(1):e0164644. doi: 10.1371/journal.pone.0164644 (PMC5218574; doi:10.1371/journal.pone.0164644)

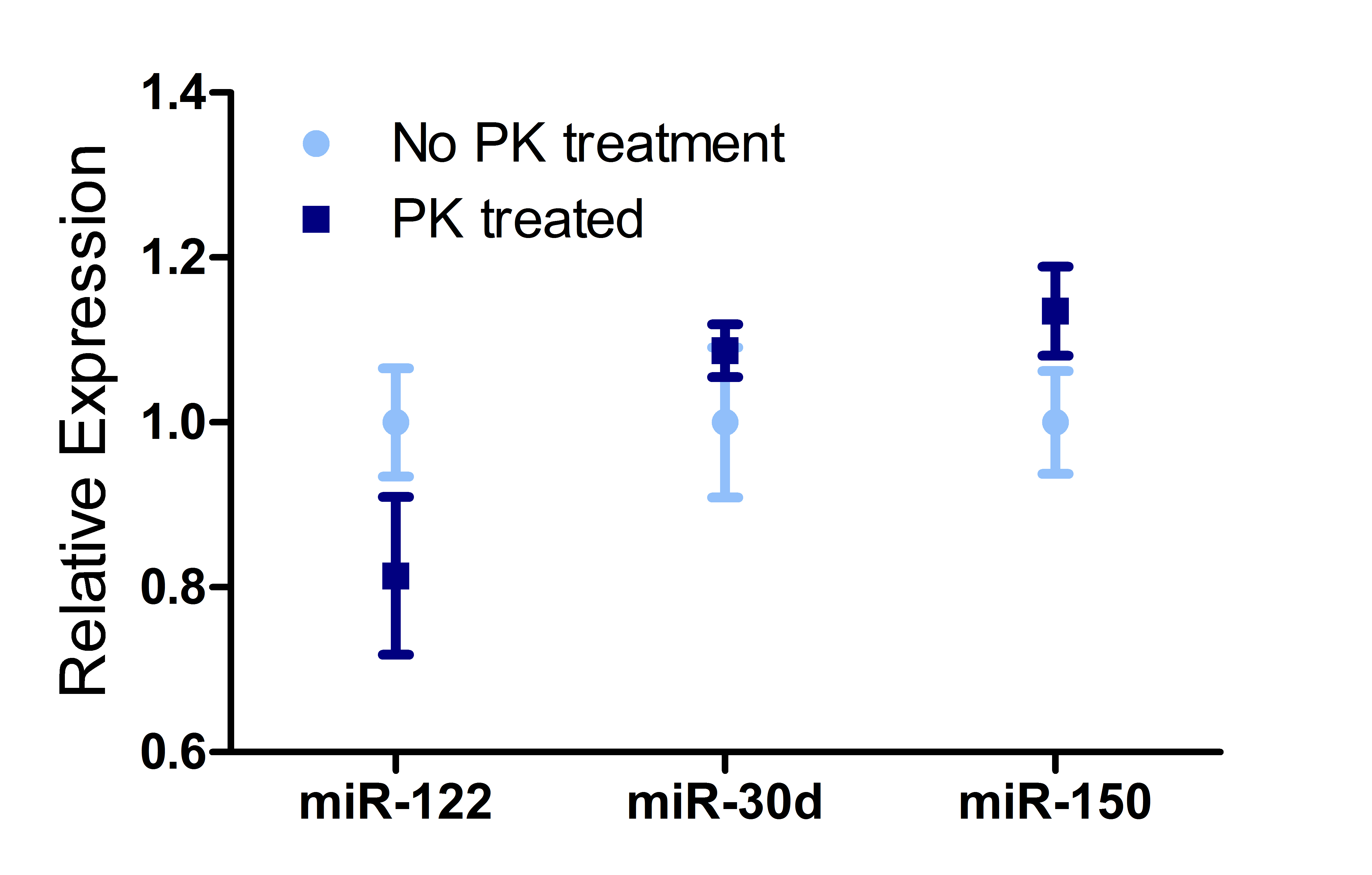

Supplement: S1 Fig — (TIF) [file pone.0164644.s005.tif]

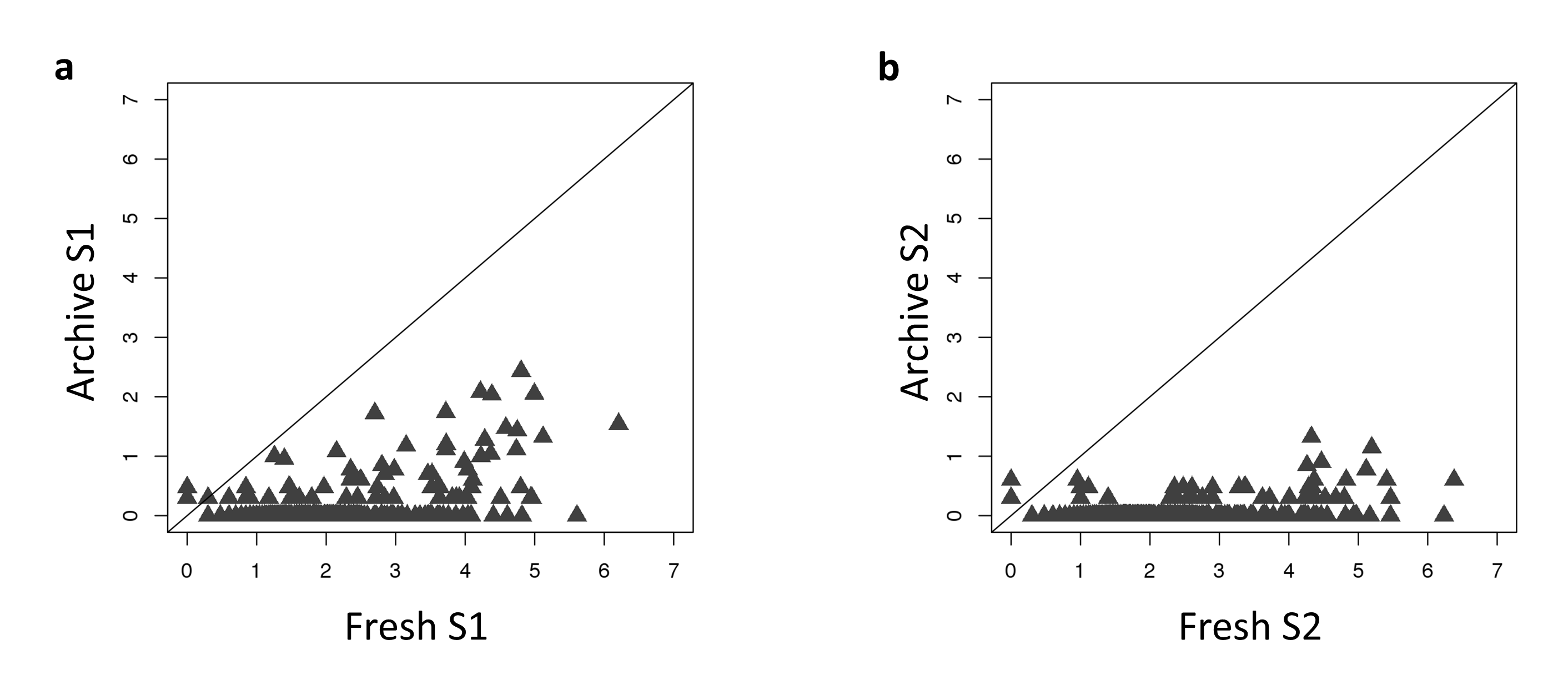

Supplement: S2 Fig — (TIF) [file pone.0164644.s006.tif]

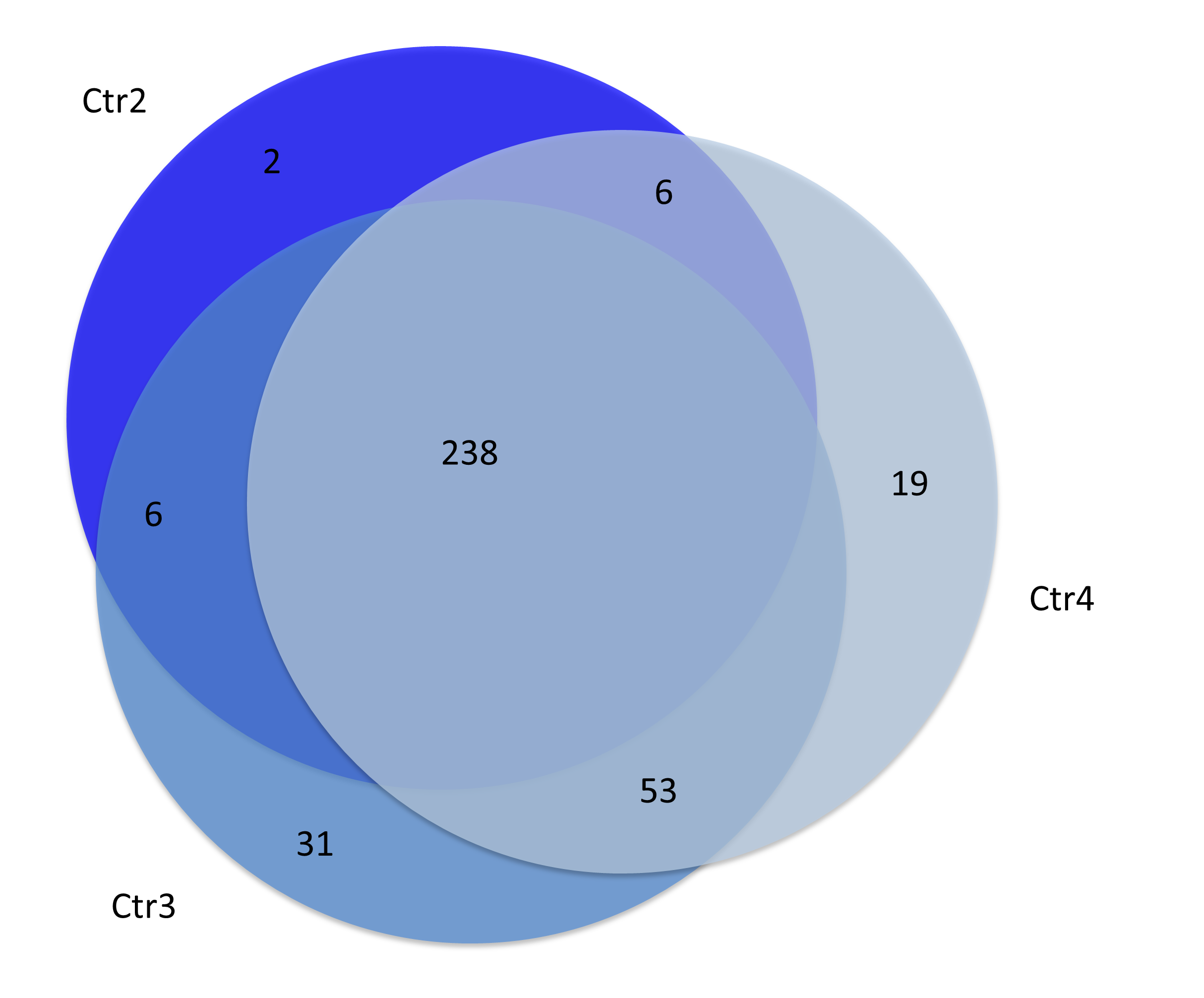

Supplement: S3 Fig — (TIF) [file pone.0164644.s007.tif]
